# Dexmedetomidine attenuates oxygen-glucose deprivation/ reperfusion-induced inflammation through the miR-17-5p/ TLR4/ NF-κB axis

**DOI:** 10.1186/s12871-022-01661-1

**Published:** 2022-04-29

**Authors:** Liangyuan Suo, Mingyu Wang

**Affiliations:** Department of Anesthesiology, Cancer Hospital of China Medical University, Liaoning Cancer Hospital, No.44 Xiaoheyan road, Dadong district, Shenyang, 110042 Liaoning China

**Keywords:** Dexmedetomidine, Oxygen-glucose deprivation/ reperfusion, Inflammation, Apoptosis, miR-17-5p, TLR4 /NF-κB signaling

## Abstract

**Background:**

Dexmedetomidine (DEX) is a selective agonist of α2-adrenergic receptors with anesthetic activity and neuroprotective benefits. However, its mechanism of action at the molecular level remains poorly defined. In this study, we investigated the protective effects of DEX on oxygen-glucose deprivation/ reperfusion (OGD/R)-induced neuronal apoptosis in PC12 cells, and evaluated its underlying mechanism(s) of neuroprotection and anti-inflammation.

**Methods:**

An OGD/R model in PC12 cells was established. PC12 cells were cultured and divided into control, OGD/R, and OGD/R + DEX (1 μM, 10 μM, 50 μM) groups. Cell apoptosis was analyzed by flow cytometry and expression profiles were determined by qRT-PCR, western blot analysis, and enzyme linked immunosorbent assays (ELISA). The interaction between miRNA and its downstream targets was evaluated through luciferase reporter assays.

**Results:**

DEX significantly decreased apoptosis rates and inhibited interleukin 1 beta (IL-1β), tumor necrosis factor alpha (TNF-α), and interleukin 6 (IL-6) release (*P* < 0.05). While expression of the pro-apoptotic proteins Bax and Caspase-3 was down-regulated, expression of Bcl-2 was upregulated in a dose-dependent manner (*P* < 0.05). Interestingly, miR-17-5p expression was down-regulated in the OGD/R group (compared to controls). Toll-like receptor 4 (TLR4), a key regulator of nuclear factor kappa-B (NF-κB) signaling, was identified as a novel target of miR-17-5p in PC12 cells. miR-17-5p expression was upregulated in the OGD/R + DEX group, suppressing TLR4 expression and reducing the secretion of proinflammatory cytokines.

**Conclusion:**

DEX inhibits OGD/R-induced inflammation and apoptosis in PC12 cells by increasing miR-17-5p expression, downregulating TLR4, and inhibiting NF-κB signaling.

## Background

Stroke causes significant disabilities and cognitive impairment in afflicted individuals throughout the world [1]. The basic pathophysiology of cerebral ischemic stroke is complex, involving the interplay of autophagy, apoptosis, oxidative stress, inflammation, and energy attenuation [2, 3]. Recently, evidence has been presented highlighting the role of miRNAs in cerebral ischemia-reperfusion injury, identifying miRNAs as potential therapeutic targets [4, 5].

Dexmedetomidine (DEX) is a α2-adrenoceptor agonist that exhibits sedative, anxiolytic, and analgesic functions [6]. DEX is known to exert positive effects (in comparison with other sedatives), including mitigation of respiratory depression and hypotension, alleviation of lung and kidney damage, and decreased neuronal apoptosis [7]. DEX also has a long-term neuroprotective influence on cognitive dysfunction and brain injury [8].

The role of microRNAs (miRs), short non-coding RNA molecules, is to bind to mRNAs and inhibit the expression of target genes. The downregulation of miRs in neuronal cells is intricately linked to neurodegenerative disease [9]. Approximately 70% of all known miRs are expressed in the brain (either locally or tissue wide), and these are critical to the functionality of the nervous system [10]. Oxygen-glucose deprivation/reperfusion (OGD/R) miRs are reported to suppress apoptosis of developing hippocampal astrocytes in rodents, thus affording protection against hepatic ischemia/ reperfusion injury [11, 12]. An interaction between miR-223-3p and TIAL1 has been demonstrated to contribute to the neuroprotective effects of DEX in hippocampal neuronal cells *in vitro* [13]. Hence, DEX may regulate OGD/R-induced inflammation and apoptosis through miRs. Hao *et al*. (2017) report that miR-17-5p is pro-apoptotic, and that miR-17-5p overexpression induces neuronal death and apoptosis [14]. However, miR-17-5p has also been reported to inhibit neuronal apoptosis and epileptiform discharge in hippocampal neurons following seizures [15].

Thus far, the overall effects of DEX on OGD/R-induced inflammation and apoptosis remain unclear. In the present study, we investigated the hypothesis that DEX-treatment suppresses OGD/R-induced inflammation and apoptosis, and we explored the potential biological mechanisms mediating these protective effects.

## Methods

### Cell lines and cell culture

PC12 cells obtained from the American Type Culture Collection (ATCC; USA) were cultured in RPMI 1640 containing 10% fetal bovine serum at 37 °C in a humidified incubator containing 5% CO_2_. Cells were passaged after reaching ~80% confluency, and seeded into 96-well plates at a density of 10^4^/mL.

Six treatment groups were established: (1) normal control group; (2) model group; (3) solvent group; (4) dexmedetomidine (DEX) low group; (5) DEX medium group; and (6) DEX high group. All treatments were performed in triplicate. In the model DEX groups, cells were cultured in glucose-free RPMI 1640 containing 30 mmol/L NaS_2_O_4_. Cells were treated with DEX (in normal saline) at 1 μmol/L, 10 μmol/L, or 50 μmol/L (an equal volume of saline was added to the control group). The cells in each group were then cultured at 37 °C for 4 h.

### miRNAs, plasmids, and cell transfections

TLR4 overexpression plasmids (pcDNA3.1) and corresponding controls (pcDNA3.1) were generated by GenePharma (Shanghai, China). miR-17-5p mimics, inhibitors, and mimic/ inhibitor negative controls (mimic NC and inhibitor NC) were generated by Ribobio (Guangzhou, China). PC12 cells were transfected with LiRNAfectamine 3000 (Invitrogen, Carlsbad, USA) according to the manufacturer’s recommendations. Cells were analyzed 24 h post-transfection.

### ELISA assays for the determination of interleukin 1 beta (IL-1β), tumor necrosis factor alpha (TNF-α), and interleukin 6 (IL-6) levels

Commercially available ELISA kits (R&D Systems, Minneapolis, USA) were used to determine the expression levels of IL-1β (#DLB50), TNF-α (#DTA00D), and IL-6 (#DR600). All ELISA kits were used as per the manufacturer’s recommendations.

### RNA extraction and qRT-PCR analysis

Total RNA was harvested using commercially available RNA extraction kits (Takara, China) according to the manufacturer’s instructions. miR-17-5p was reverse transcribed to cDNA using the MicroRNA Reverse Transcription Kit (Thermo Fisher Scientific). Real-time PCR amplifications were performed on an ABI7900 Fast Real-Time PCR System using SYBR Green Real-Time PCR Master Mix (Thermo Fisher Scientific). U6 and GAPDH served as internal controls for miR-17-5p and mRNA expression, respectively. The comparative Ct method was used to calculate relative gene expression levels.

### Luciferase reporter assays

The TargetScan V7.2 database was used to assess putative binding sites for miR-17-5p in the TLR4 3′-UTR. Wild-type (WT) fragments of TLR4 3-'UTR were amplified from genomic DNA and subcloned into a pmirGLO reporter vector (Promega, Madison, USA). This construct was termed TLR4 3′-UTR-WT. TLR4 3-'UTR mutations were performed through site-directed mutagenesis kit (Stratagene, San Diego, USA). The construct was termed TLR4 3′-UTR-MUT. For luciferase activity, HEK293 cells were transfected with miRNAs (miR mimic or NC mimic) and luciferase reporter vectors (TLR4 3′-UTR-WT or TLR4 3′-UTR-MUT) using Lipofectamine 3000 reagent (Invitrogen). Forty-eight hours post-transfection, luciferase activity was assessed using the Dual-Luciferase® Reporter (DLR™) Assay System (Promega).

### Western blot analysis

Cells were lysed in ice-cold radioimmunoprecipitation assay buffer (Roche, Basel, Switzerland) supplemented with protease inhibitors. Protein concentrations were determined using dioctanoic acid assays (Thermo Fisher Scientific). Equal volumes of proteins were separated on 10% sodium lauryl sulfate-polyacrylamide gels. Separated proteins were transferred to polyvinylidene fluoride membranes and blocked in 1.5% skimmed milk in Tris buffered saline containing Tween 20 (TBST). Membranes were probed with primary antibodies at 4 °C overnight and washed in TBST. The membranes were then probed with horseradish peroxidase-conjugated secondary antibodies (Cell Signaling Technology) for 2 h at room temperature. ECL kits (Thermo Fisher Scientific) were used to determine band intensities on the membranes as per the manufacturer’s recommendations. The primary antibodies were TLR4, p-p65, p65, p-IκBα, IκBα, Blc-2, Bax, and caspase-3 (Cell Signaling, Danfoss, Mass.) GAPDH (Santa Cruz, California) was also probed as a loading control.

### Apoptosis assays

After 48 h of H_2_O_2_ treatment, cells were stained with 5 μL Annexin V-FITC and 5 μL PI (BD Biosciences, USA). Apoptosis was then analyzed by flow cytometry (Becton Dickinson, USA). Annexin V-positive cell populations were considered apoptotic.

### Statistical analysis

Statistical analysis was performed using SPSS 18.0 software. All data were tested for normality and homogeneity of variance, and those that met this criterion were expressed as mean ± standard deviation of the mean (SD). The differences between two groups were evaluated by Student’s t-test, while differences between three or more groups were analyzed by ANOVA (with Bonferroni Multiple Comparison tests). Data that did not meet the criterion for normality or homogeneity of variance were transformed into categorical variables and analyzed by Mann-Whitney U tests. The data were graphed using GraphPad Prism Software (version 5, GraphPad Software, Inc., La Jolla, CA). A *P* < 0.05 was used to test for statistical significance.

## Results

### Dexmedetomidine attenuates OGD/R-induced inflammation and apoptosis in PC12 cells

**Fig. 1:**
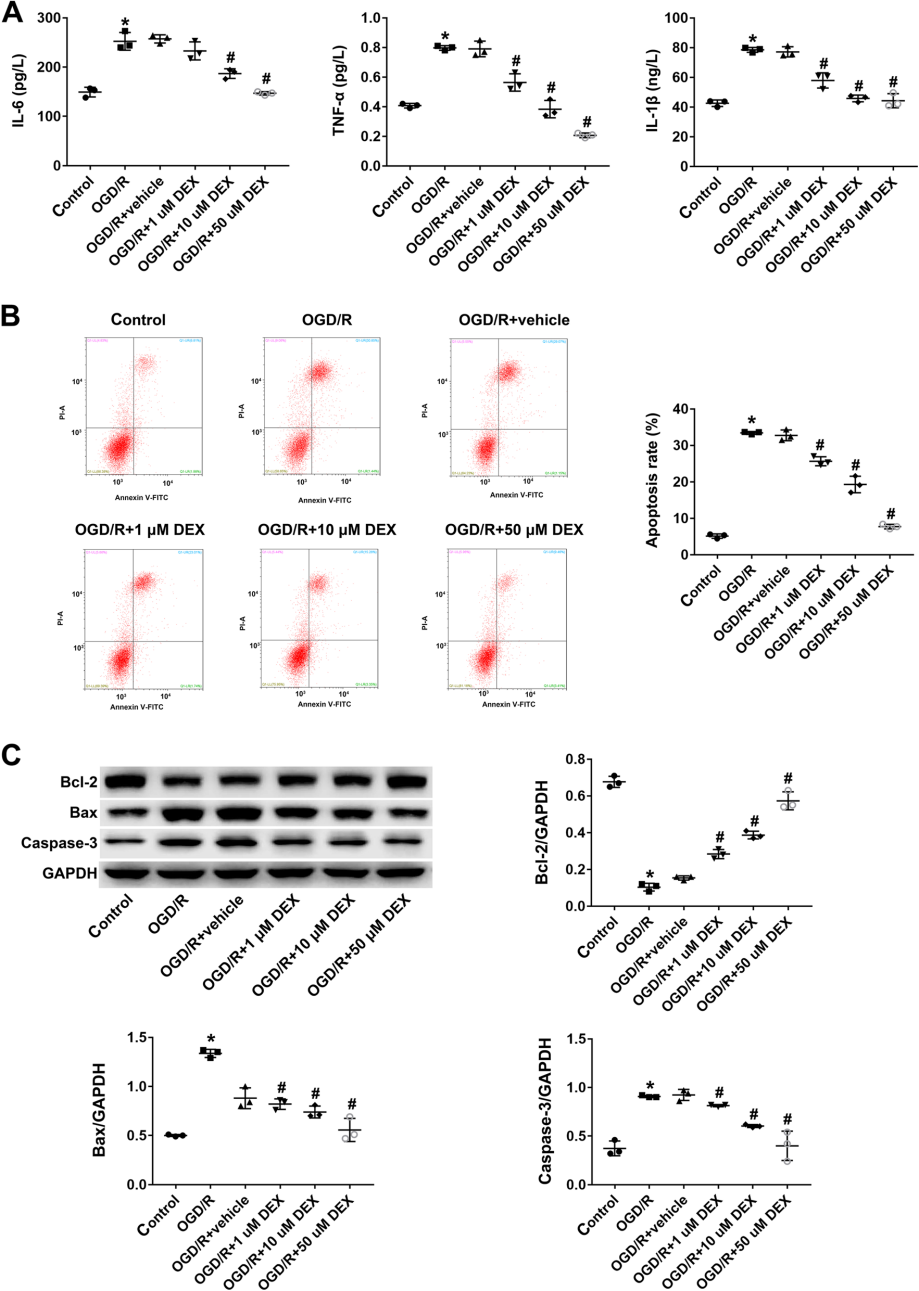
Dexmedetomidine attenuates OGD/R-induced inflammation and apoptosis in PC12 cells. **A** ELISA-based detection of IL-1β, TNF-α, and IL-6 production in PC12 cells. **B** Apoptosis rates detected by flow cytometry in H_2_O_2_-treated PC12 cells. **C** Expression of Bcl-2, Bax, and caspase 3 detected by western analysis. Data represent the mean ± SD of three independent experiments. *P < 0.05 vs control; #*P* < 0.05 vs OGD/R + vehicle

The overexpression of inflammatory mediators, including IL-6, IL-1β, and TNF-α, is linked to OGD/R. Thus, IL-6, IL-1β, and TNF-α expression levels were higher in the OGD/R group Compared to the control, DEX treatment suppressed IL-6, IL-1β, and TNF-α expression levels in a dose-dependent manner (*P* < 0.05; Fig. 1A). Flow cytometry analysis revealed that OGD/R treatment increased the rate of apoptosis in PC12 cells, whilst DEX treatment significantly suppressed OGD/R-induced apoptosis (*P* < 0.05; Fig. 1B). DEX treatment increased the expression of the anti-apoptotic protein Bcl-2, and reduced the expression of the pro-apoptotic proteins Bcl-2-associated X protein (Bax) and Caspase-3 (*P* < 0.05; Fig. 1C). These effects in the OGD/R + DEX group were dose-dependent. Together, these results provide evidence that DEX inhibits apoptosis in OGD/R -treated PC12 cells.

### Dexmedetomidine inhibits TLR4/ NF-κB signaling through miR-17-5p in PC12 cells

**Fig. 2:**
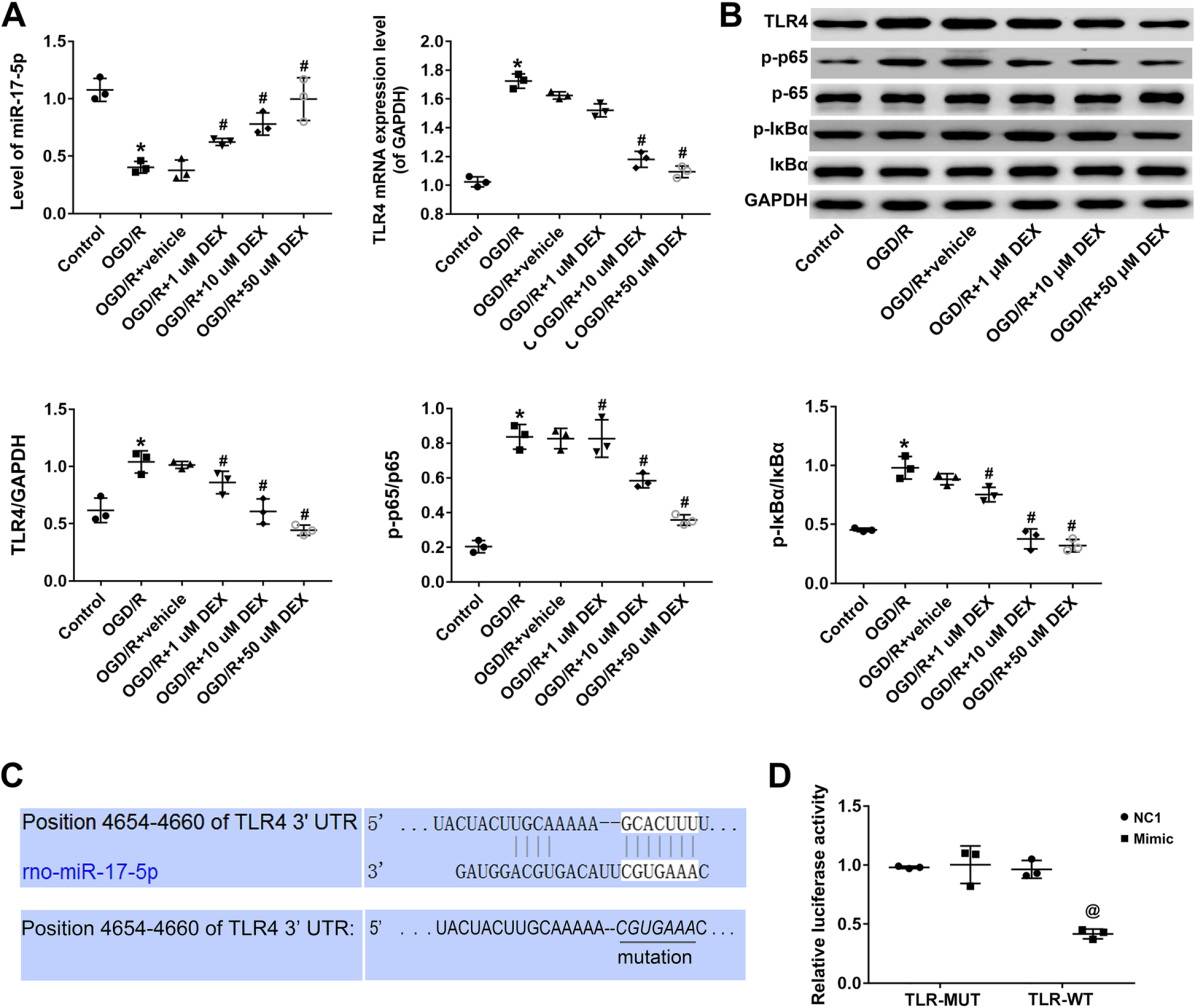
Dexmedetomidine inhibits TLR4/ NF-κB signaling via miR-17-5p in PC12 cells. **A** miR-17-5p and TLR4 expression in treatment and control groups determined by qRT-PCR. **B** Relative expression of TLR4, p-IκBα, IκBα, p-p65, and p65 in each group detected by western analysis. Left, protein band; right, relative protein expression level. **C** Putative binding sites for miR-17-5p in the TLR4 3′-UTR. **D** Luciferase activities of TLR4 3′-UTR-WT and TLR4 3′-UTR-MUT constructs in HEK293 cells following treatment with NC or miR-17-5p mimic determined using the Dual-Luciferase Reporter Assay system. Data represent the mean ± SD of three independent experiments. *P < 0.05 vs control; #*P* < 0.05 vs OGD/R + vehicle

miR-17-5p was significantly down-regulated in PC12 cells in the OGD/R group (compared to the control group). In comparison to the OGD/R + vehicle group, DEX treatment upregulated miR-17-5p in a dose-dependent manner (*P* < 0.05; Fig. 2A). Because TLR4 is known to regulate inflammatory response through NF-κB signaling, TLR4/ NF-κB signaling was further examined. Western blot analysis revealed that DEX treatment significantly decreased the levels of p-IκBα and p-p65 proteins, but not overall IκBα and p65 expression levels. A reduction in TLR4 expression induced a decrease in the levels of p-IκBα and p-p65 (*P* < 0.05; Fig. 2B). The predicted binding sites for miR-17-5p in the 3′-UTR of target genes were analyzed using TargetScan, and the TLR4 3′-UTR (position 4654–4660) was shown to possess complementary binding sites for miR-17-5p (*P* < 0.05; Fig. 2C). The WT and MUT TLR4 3′-UTR regions were subcloned into luciferase reporter vectors, and the resulting luciferase activities in HEK293 cells co-transfected with miRNAs and luciferase reporter vectors were determined. miR-17-5p overexpression repressed the luciferase activity of TLR4 3′-UTR-WT, but had no effect on the activity of TLR4 3′-UTR-MUT in HEK293 cells (*P* < 0.05; Fig. 2D).

### miR-17-5p inhibits TLR4/ NF-κB signaling, inflammation, and apoptosis in PC12 cells following OGD/R

**Fig. 3:**
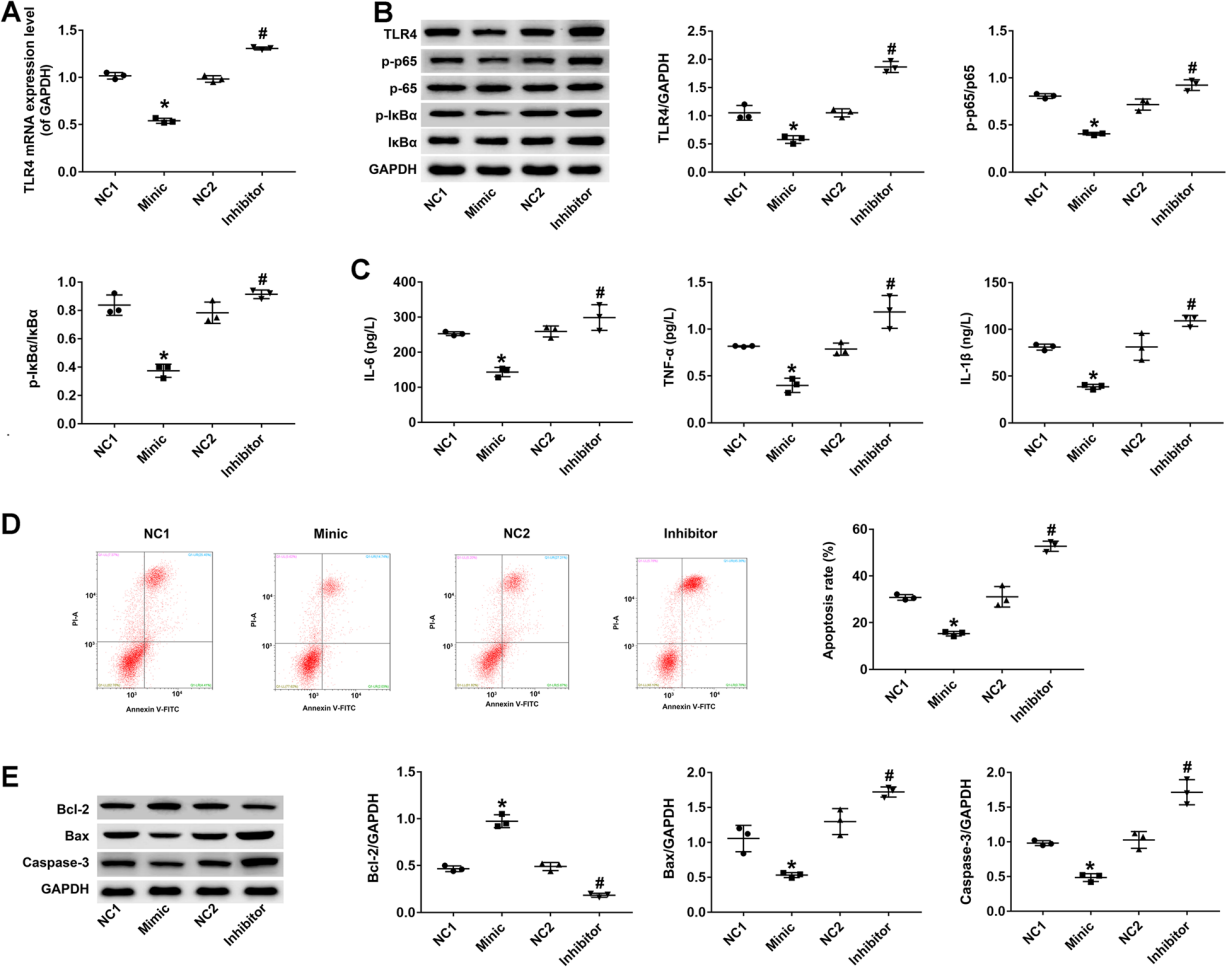
miR-17-5p inhibits TLR4/ NF-κB signaling, inflammation, and apoptosis in PC12 cells following OGD/R. **A** TLR4 expression in treatment and control groups determined by qRT-PCR. **B** Relative expression of p-IκBα, IκBα, p-p65, and p65 in PC12 cells detected by western analysis after transfection with miR-17-5p mimic or miR-17-5p inhibitor. NC1, negative vector control for miR-17-5p mimic; NC2, negative vector control for miR-17-5p. **C** PC12 cells were transfected with miR-17-5p mimic and miR-17-5p inhibitor for 48 h, then TNF-α, IL-6, and IL-1β levels were measured by ELISA. **D** Apoptosis rate was detected by flow cytometry in H_2_O_2_-treated PC12 cells after transfection with miR-17-5p mimic or miR-17-5p inhibitor. **E** Relative expression of Bcl-2, Bax, and caspase 3 detected by western analysis after transfection with miR-17-5p mimic or miR-17-5p inhibitor. Data represent the mean ± SD of three independent experiments. *P < 0.05 vs control; #*P* < 0.05 vs OGD/R + vehicle

Because TLR4 regulates inflammatory responses via NF-κB signaling, the interaction between miR-17-5p and TLR4/ NF-κB was examined. Western blot analysis revealed that miR-17-5p overexpression significantly decreased the levels of TLR4, p-IκBα, and p-p65 proteins, but not overall IκBα and p65 expression levels. In contrast, miR-17-5p silencing increased the levels of TLR4, p-IκBα, and p-p65 proteins (*P* < 0.05; Fig. 3A). As miR-17-5p was down-regulated in the OGD/R group, the effects of miR-17-5p overexpression on the production of pro-inflammatory cytokines in PC12 cells were also determined. ELISA analysis demonstrated that the levels of IL-1β, TNF-α, and IL-6 proteins in PC12 cells overexpressing miR-17-5p were significantly decreased (compared with those transfected with NC mimic). In contrast, knockdown of miR-17-5p enhanced the production of pro-inflammatory cytokines in PC12 cells (*P* < 0.05; Fig. 3B). Next, we examined the rate of apoptosis in PC12 cells transfected with miR-17-5p mimic after H_2_O_2_ stimulation. Flow cytometry analysis revealed that overexpression of miR-17-5p significantly suppressed H_2_O_2_-induced apoptosis in PC12 cells, increased Bcl-2 levels, and suppressed Bax and Caspase-3 expression. As expected, miR-17-5p silencing produced the opposite phenotype (*P* < 0.05; Fig. 3C, D).

### TLR4 overexpression and miR-17-5p silencing inhibit DEX-induced anti-inflammatory and anti-apoptotic phenotypes in PC12 cells following OGD/R

**Fig. 4:**
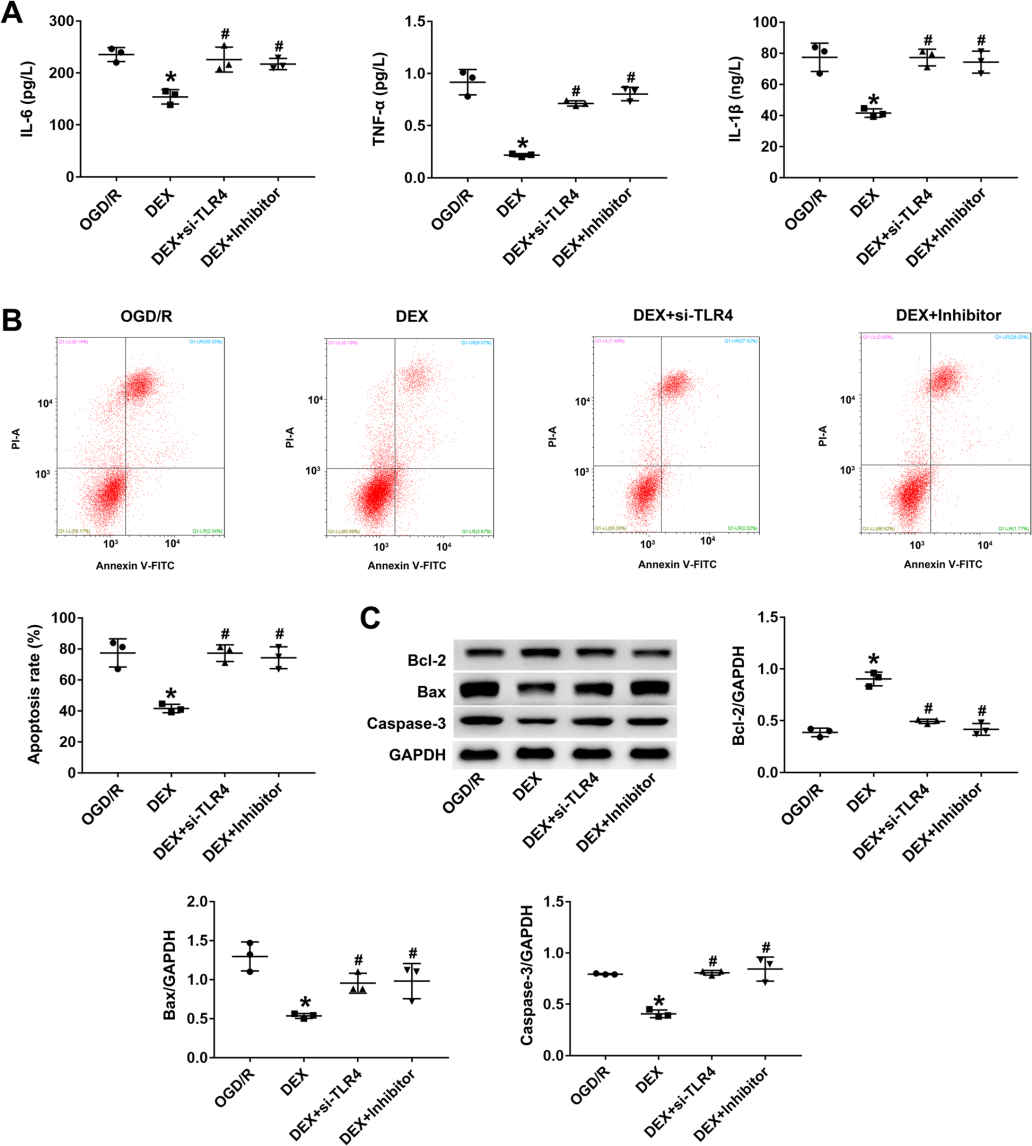
TLR4 overexpression and miR-17-5p silencing inhibit the anti-inflammatory and anti-apoptotic effects of DEX in PC12 cells following OGD/R. **A** PC12 cells were transfected with pcDNA3.1-TLR4 and miR-17-5p inhibitor, and TNF-α, IL-6, and IL-1β levels were measured by ELISA. **B** Apoptosis rate was detected by flow cytometry in H_2_O_2_-treated PC12 cells after transfection with pcDNA3.1-TLR4 and miR-17-5p inhibitor. **C** Relative expression of Bcl-2, Bax, and caspase 3 in PC12 cells transfected with pcDNA3.1-TLR4 and miR-17-5p inhibitor detected by western blot. Data are the mean ± SD of three independent experiments. *P < 0.05 vs OGD/R; #*P* < 0.05 vs DEX

Rescue experiments were performed to determine if the combined overexpression of TLR4 and miR-17-5p silencing could attenuate the effects of DEX treatment on OGD/R-induced inflammation and apoptosis in PC12 cells. TLR4 overexpression and miR-17-5p knockdown markedly increased the production of IL-1β, TNF-α, and IL-6 proteins in PC12 cells compared to the DEX group (*P* < 0.05; Fig. 4A). Furthermore, TLR4 overexpression and miR-17-5p silencing significantly increased the rate of apoptosis in PC12 cells, decreased the expression of Bcl-2, and increased Bax and Caspase-3 expression (*P* < 0.05; Fig. 4B, C).

## Discussion

The mechanism(s) of cerebral ischemia-reperfusion injury are complex, involving excitatory amino acid toxicity, oxidative stress, and inflammatory responses [16]. These factors are interconnected and ultimately induce apoptotic signaling and programmed neuronal cell death [17]. Apoptosis after cerebral ischemia-reperfusion injury is a major form of neuronal death [18]. Inhibition of apoptosis can be used as a potential therapeutic intervention for cerebral ischemia-reperfusion injury. In this study, an OGD/R cell model of PC12 cells was established to simulate cerebral ischemia-reperfusion injury *in vitro*, and to explore the neuroprotective effects of DEX. Li *et al*. (2016) report that DEX is 1600-fold more selective for α2 over α1, inhibiting apoptosis and exerting neuroprotective effects in the developing brain [19]. DEX preconditioning is reported to protect the heart from apoptosis following ischemic/ reperfusion injury in a diabetic rat model (both *in vivo* and *in vitro*) by activating PI3K/ Akt signaling [20]. Furthermore, DEX preconditioning protects the heart from apoptosis in ischemic/ reperfusion injury in diabetic rats by activating PI3K/ Akt signaling *in vivo* and *in vitro* [21]. However, the mechanisms by which DEX regulates these effects remain to be elucidated.

miRNAs participate in a range of essential biological processes, including neuronal apoptosis during ischemic stroke and nervous system dysfunction [22]. Here, we explored the underlying biological mechanism of DEX attenuation of OGD/R-induced neurotoxicity, and we investigated the involvement of miR-17-5p and potential molecular factors. miR-17-5p is significantly upregulated in the early stage of cerebral ischemia-reperfusion injury (within 4 h). Moreover, onset of miR-17-5p upregulation is earlier than the observed changes in urea nitrogen level and neutrophil gelatinase-related lipocalin (NGAL) concentration [23]. miR-17-5p is known to be induced by p53 and to protect from renal ischemia-reperfusion injury by targeting death receptor 6 [24]. In addition, miR-17-5p is down-regulated by the Act A/ Smads signaling loop, thus enhancing the neuroprotective effect after ischemic injury [25]. Although these effects of miR-17-5p may modulate the therapeutic efficacy of DEX on OGD/R-induced neurotoxicity, no reports to this effect have been published thus far.

TLR4 belongs to the Toll-like receptor family. These innate pattern recognition receptors mediate the host response to pathogen infection [26]. TLR4 activation promotes the production of inflammatory cytokines, such as IL-1β, TNF-α, and IL-6 [27]. Aberrant IL-1β and IL-6 responses induced by TLR4 have been observed in patients. TLR4 mRNA is reported to have a binding site for miR-17-5p [28]. Thus, miR-17-5p may regulate inflammation induced by oxygen and glucose deprivation/ reperfusion via TLR4/ NF-κB [29].

In our present study, we report that miR-17-5p was downregulated in the OGD/R group, and that miR-17-5p mediated OGD/R-induced inflammation and apoptosis. DEX treatment increased miR-17-5p expression in a dose-dependent manner in PC12 cells. Moreover, miR-17-5p overexpression suppressed the inflammatory response by inhibiting NF-κB. Conversely, miR-17-5p downregulation produced the opposite phenotype. To explore the mechanisms underlying these effects, the TargetScan V7.2 database was employed to identify miR-17-5p regulated genes. miR-17-5p was predicted to bind to TLR4 mRNA. Our results reveal that miR-17-5p levels were negatively correlated with TLR4 levels, and that miR-17-5p binding to the 3′-UTR of TLR4 suppressed expression in a luciferase gene reporter assay. However, the role of miR-17-5p remained undefined.

To further investigate the role of miR-17-5p in OGD/R-induced inflammation and apoptosis in PC12 cells, miR-17-5p mimic and miR-17-5p inhibitor were separately transfected into each group. miR-17-5p mimic was observed to inhibit OGD/R-induced inflammation and apoptosis. Moreover, we identified NF-κB signaling as a potential mediator of miR-17-5p inhibition, and we subsequently demonstrated that miR-17-5p mimic could inhibit TLR4/ NF-κB signaling. For many drugs such as soy isoflavones and genistein, phosphorylation of IκBα and P65 is necessary for their neuroprotective effects [30]. From a consideration of these results, we propose that DEX upregulates miR-17-5p, and that miR-17-5p inhibits NF-κB subsequently reducing OGD/R-induced inflammation and apoptosis. In addition, we observed that inhibition of OGD/R-induced inflammation and apoptosis was suppressed following TLR4 overexpression or miR-17-5p silencing, which suggests that DEX attenuates oxygen-glucose deprivation/ reperfusion-induced inflammation and apoptosis in PC12 cells through an effect on the miR-17-5p/ TLR4/ NF-κB axis. Together, our data demonstrate the potential of DEX as a novel intervention strategy for cerebral ischemia-reperfusion injury. These findings require further verification in human stroke patients.

Our study had several limitations worth noting. First, the optimal concentration of DEX *in vivo* was not investigated. Therefore, DEX dosage should be further investigated in *in vivo* experiments. Second, DEX has only been observed thus far to up-regulate the expression of miR-17-5p in PC12 cells. Further studies are required to confirm these effects *in vivo*. On the side, in this study, DEX mainly exerts its protective effects through its anti-inflammatory activity, which is an ancillary activity compared to its main property (hypnotic drug). Would glucocorticoids, the reference anti-inflammatory drugs, provide the same protection while avoiding undesired central effects? Several studies in recent years have reported on this, Wang et al. [31] found knockout in studies glucocorticoid-regulated kinase 1 (SGK1) knockdown upregulated beclin-1 and LC-3 expression mediated by Intracarotid cold saline infusion (ICSI), This suggests that ICSI has a neuroprotective effect on ischemic stroke after reperfusion through up-regulation of SGK1 and inhibition of autophagy. The role of glucocorticoid signaling was also reported in the study of Perović et al. [32], They found Food restriction (FR) applied prior to TBI significantly changes p-GR levels, and it’s transcriptional activity during the recovery period after TBI. Moreover, as a pretreatment, FR modulates other protective factors in response to TBI, such as 11β-HSD1, NF-κB (p65) and HSP70 that act in parallel with GR in it’s anti-inflammatory and neuroprotective effects in the rat model of brain injury. That’s what we’re going to do next. Conclusion.

DEX inhibits OGD/R-induced inflammation and apoptosis in PC12 cells by increasing miR-17-5p expression, downregulating TLR4, and inhibiting NF-κB signaling. These results preliminarily explain the neuroprotective mechanism of DEX in ischemic stroke, and provide a direction for further searching for therapeutic targets for ischemic stroke.

## Data Availability

The datasets used and/or analyzed during the current study are available from the corresponding author upon reasonable request. The authors declare that there was no conflict of interest in this paper.

## Abbreviations

DEX: Dexmedetomidine
ELISA: Enzyme Linked Immunosorbent Assays
miRs: microRNAs
mimic NC: Mimic Negative Controls
inhibitor NC: Inhibitor Negative Controls
IL-1β: Interleukin 1 beta
TNF-α: Tumor Necrosis Factor-alpha
IL-6: Interleukin 6
WT: Wild-type
TBST: Tris Buffered Saline containing Tween 20
SD: Standard Deviation of the mean
Bax: Bcl-2 associated X protein
Caspase-3: Cysteinyl aspartate specific proteinase-3

## References

[1] Huang XP, Ding H, Yang XQ (2017). Synergism and mechanism of Astragaloside IV combined with Ginsenoside Rg1 against autophagic injury of PC12 cells induced by oxygen glucose deprivation/reoxygenation. Biomed Pharmacother.

[2] Papanagiotou P, White CJ (2016). Endovascular reperfusion strategies for acute stroke. JACC Cardiovasc Interv.

[3] Schaller B, Graf R (2004). Cerebral ischemia and reperfusion: the pathophysiologic concept as a basis for clinical therapy. J Cereb Blood Flow Metab.

[4] Ogata K, Sumida K, Miyata K, Kushida M, Kuwamura M, Yamate J (2015). Circulating miR-9* and miR-384-5p as potential indicators for trimethyltin-induced neurotoxicity. Toxicol Pathol.

[5] Kosik KS. The neuronal microRNA system. Nat Rev Neurosci. 2006;7(12):911–20. 10.1038/nrn2037 17115073

[6] Alam A, Suen KC, Hana Z, Sanders RD, Maze M, Ma D (2017). Neuroprotection and neurotoxicity in the developing brain: an update on the effects of dexmedetomidine and xenon. Neurotoxicol Teratol.

[7] Endesfelder S, Makki H, von Haefen C, Spies CD, Bührer C, Sifringer M (2017). Neuroprotective effects of dexmedetomidine against hyperoxia-induced injury in the developing rat brain. PLoS One.

[8] Degos V, Charpentier TL, Chhor V (2013). Neuroprotective effects of dexmedetomidine against glutamate agonist-induced neuronal cell death are related to increased astrocyte brain-derived neurotrophic factor expression. Anesthesiology..

[9] Ding L, Zhang H, Mi W (2015). Zhong Nan Da Xue Xue Bao Yi Xue Ban.

[10] Paeschke N, von Haefen C, Endesfelder S, Sifringer M, Spies CD (2017). Dexmedetomidine Prevents Lipopolysaccharide-Induced MicroRNA Expression in the Adult Rat Brain. Int J Mol Sci.

[11] Zhang L, Dong LY, Li YJ, Hong Z, Wei WS (2012). The microRNA miR-181c controls microglia-mediated neuronal apoptosis by suppressing tumor necrosis factor. J Neuroinflammation.

[12] Sun ZZ, Lv ZY, Tian WJ, Yang Y (2017). MicroRNA-132 protects hippocampal neurons against oxygen-glucose deprivation-induced apoptosis. Int J Immunopathol Pharmacol.

[13] Sun WC, Liang ZD, Pei L (2015). Propofol-induced rno-miR-665 targets BCL2L1 and influences apoptosis in rodent developing hippocampal astrocytes. Neurotoxicology..

[14] Hao W, Zhao ZH, Meng QT, Tie ME, Lei SQ, Xia ZY (2017). Propofol protects against hepatic ischemia/reperfusion injury via miR-133a-5p regulating the expression of MAPK6. Cell Biol Int.

[15] Wang Q, Yu H, Yu H, Ma M, Ma Y, Li R (2019). miR-223-3p/TIAL1 interaction is involved in the mechanisms associated with the neuroprotective effects of dexmedetomidine on hippocampal neuronal cells *in vitro*. Mol Med Rep.

[16] Wang JX, Jia XJ, Liu Y (2020). Silencing of miR-17-5p suppresses cell proliferation and promotes cell apoptosis by directly targeting PIK3R1 in laryngeal squamous cell carcinoma. Cancer Cell Int.

[17] An JH, Chen ZY, Ma QL, Wang HJ, Zhang JQ, Shi FW (2019). LncRNA SNHG16 promoted proliferation and inflammatory response of macrophages through miR-17-5p/NF-κB signaling pathway in patients with atherosclerosis. Eur Rev Med Pharmacol Sci.

[18] Wang M, Li YJ, Ding Y (2016). Silibinin prevents autophagic cell death upon oxidative stress in cortical neurons and cerebral ischemia-reperfusion injury. Mol Neurobiol.

[19] Li C, Liu Y, Tang P (2016). Hydrogen sulfide prevents OGD/R-induced apoptosis by suppressing the phosphorylation of p38 and secretion of IL-6 in PC12 cells. Neuroreport..

[20] Chang JH, Jin M, Liu JT (2020). Dexmedetomidine pretreatment protects the heart against apoptosis in ischemia/reperfusion injury in diabetic rats by activating PI3K/Akt signaling *in vivo* and *in vitro* [J]. Biomed Pharmacother.

[21] Sun W, Zhao J, Li C (2020). Dexmedetomidine provides protection against hippocampal neuron apoptosis and cognitive impairment in mice with Alzheimer’s disease by mediating the miR-129/YAP1/JAG1 axis [J]. Mol Neurobiol.

[22] Liu R, Zhong X, Zeng J (2017). 3′-Daidzein sulfonate sodium inhibits neuronal apoptosis induced by cerebral ischemia-reperfusion. Int J Mol Med.

[23] Ma L, Wu K, Liu K (2015). Changes of miRNA-17-5p, miRNA-21 and miRNA-106a level during rat kidney ischemia-reperfusion injury [J]. Zhonghua Yi Xue Za Zhi.

[24] Hao J, Wei Q, Mei S (2017). Induction of microRNA-17-5p by p53 protects against renal ischemia-reperfusion injury by targeting death receptor 6[J]. Kidney Int.

[25] Wang JQ, Dong Y, Li SJ (2019). Knockdown of microRNA-17-5p enhances the neuroprotective effect of Act A/Smads signal loop after ischemic injury [J]. Neurochem Res.

[26] Liu B, Li F, Shi J, Yang D, Deng Y, Gong Q (2016). Gastrodin ameliorates subacute phase cerebral ischemia-reperfusion injury by inhibiting inflammation and apoptosis in rats. Mol Med Rep.

[27] Ma D, Hossain M, Rajakumaraswamy N (2004). Dexmedetomidine produces its neuroprotective effect via the alpha 2A-adrenoceptor subtype. Eur J Pharmacol.

[28] Ji ZR, Xue WL, Zhang L (2019). Schisandrin B attenuates inflammation in LPS-induced sepsis through miR-17-5p downregulating TLR4[J]. Inflammation.

[29] Suo L, Wang M (2020). Dexmedetomidine attenuates oxygen-glucose deprivation/reperfusion-induced inflammation through the miR-17-5p/TLR4/NF-κB axis [J].

[30] Huang R, Chen Y, Yu AC, Hertz L (2000). Dexmedetomidine-induced stimulation of glutamine oxidation in astrocytes: a possible mechanism for its neuroprotective activity. J Cereb Blood Flow Metab.

[31] Wang D, Huang Z, Li L (2019). Intracarotid cold saline infusion contributes to neuroprotection in MCAO-induced ischemic stroke in rats via serum and glucocorticoid-regulated kinase 1[J]. Mol Med Rep.

[32] Perović M, Jović M, Todorović S, et al. Neuroprotective effects of food restriction in a rat model of traumatic brain injury - the role of glucocorticoid signaling. Nutr Neurosci. 2022;25(3):537–49. Epub 2020. 10.1080/1028415X.2020.1769410 32476608

